# Characteristics and Outcome of FLT3-ITD-Positive Pediatric Acute Myeloid Leukemia—Experience of Polish Pediatric Leukemia and Lymphoma Study Group from 2005 to 2022

**DOI:** 10.3390/cancers15184557

**Published:** 2023-09-14

**Authors:** Małgorzata Czogała, Wojciech Czogała, Katarzyna Pawińska-Wąsikowska, Teofila Książek, Karolina Bukowska-Strakova, Barbara Sikorska-Fic, Paweł Łaguna, Anna Fałkowska, Katarzyna Drabko, Katarzyna Muszyńska-Rosłan, Maryna Krawczuk-Rybak, Marta Kozłowska, Ninela Irga-Jaworska, Karolina Zielezińska, Tomasz Urasiński, Natalia Bartoszewicz, Jan Styczyński, Jolanta Skalska-Sadowska, Jacek Wachowiak, Anna Rodziewicz-Konarska, Krzysztof Kałwak, Małgorzata Ciebiera, Radosław Chaber, Agnieszka Mizia-Malarz, Agnieszka Chodała-Grzywacz, Grażyna Karolczyk, Katarzyna Bobeff, Wojciech Młynarski, Katarzyna Mycko, Wanda Badowska, Renata Tomaszewska, Tomasz Szczepański, Katarzyna Machnik, Natalia Zamorska, Walentyna Balwierz, Szymon Skoczeń

**Affiliations:** 1 Department of Pediatric Oncology and Hematology, Institute of Pediatrics, Jagiellonian University Medical College, 30-663 Krakow, Poland; wojciech.czogala@uj.edu.pl (W.C.); katarzyna.pawinska-wasikowska@uj.edu.pl (K.P.-W.); walentyna.balwierz@uj.edu.pl (W.B.); szymon.skoczen@uj.edu.pl (S.S.); 2 Department of Pediatric Oncology and Hematology, University Children Hospital, 30-683 Krakow, Poland; teofila.ksiazek@uj.edu.pl; 3 Department of Medical Genetics, Institute of Pediatrics, Jagiellonian University Medical College, 30-663 Krakow, Poland; 4 Department of Clinical Immunology, Institute of Pediatrics, Jagiellonian University Medical College, 30-663 Krakow, Poland; k.bukowska-strakova@uj.edu.pl; 5 Department of Pediatrics, Oncology, Hematology and Transplantology, Medical University of Warsaw, 02-091 Warszawa, Poland; basiasf@poczta.onet.pl (B.S.-F.); pawel.laguna@wum.edu.pl (P.Ł.); 6 Department of Paediatric Haematology and Oncology and Transplantology, Medical University of Lublin, 20-095 Lublin, Poland; ania1589@gmail.com (A.F.); katarzynadrabko@umlub.pl (K.D.); 7 Department of Pediatric Oncology and Hematology, Medical University of Bialystok, 15-089 Bialystok, Poland; katarzyna.muszynska-roslan@umb.edu.pl (K.M.-R.); rybak@umb.edu.pl (M.K.-R.); 8 Department of Pediatrics, Hematology and Oncology, Medical University of Gdansk, 80-210 Gdansk, Poland; marta.kozlowska@gumed.edu.pl (M.K.); nirga@gumed.edu.pl (N.I.-J.); 9 Department of Paediatrics, Hemato-Oncology and Gastroenterology, Pomeranian Medical University in Szczecin, 71-252 Szczecin, Poland; karolina.zielezinska@pum.edu.pl (K.Z.); urasin@pum.edu.pl (T.U.); 10 Department of Pediatric Hematology and Oncology, Collegium Medicum, Nicolaus Copernicus University Torun, Bydgoszcz, 85-094 Bydgoszcz, Poland; natalabar@wp.pl (N.B.); jstyczynski@cm.umk.pl (J.S.); 11 Department of Pediatric Oncology, Hematology and Transplantology, Poznan University of Medical Sciences, 60-572 Poznan, Poland; jsk@poczta.onet.eu (J.S.-S.); wachowiak.jacek@outlook.com (J.W.); 12 Department of Bone Marrow Transplantation, Pediatric Oncology and Hematology, Medical University of Wroclaw, 50-556 Wroclaw, Poland; anna.maria.rodziewicz@gmail.com (A.R.-K.); krzysztof.kalwak@gmail.com (K.K.); 13 Clinic of Pediatric Oncology and Hematology, State Hospital 2, 35-301 Rzeszów, Poland; malgorzata.ciebiera@gmail.com (M.C.); rchaber@ur.pl (R.C.); 14 Institute of Medical Sciences, Medical College of Rzeszow University, 35-959 Rzeszów, Poland; 15 Department of Oncology, Hematology and Chemotherapy, Upper Silesia Children’s Care Health Centre, 40-752 Katowice, Poland; a.mizia@wp.pl; 16 Department of Pediatrics, Medical University of Silesia, Upper Silesia Children’s Care Health Centre, 40-752 Katowice, Poland; 17 Department of Pediatric Hematology and Oncology, Regional Polyclinic Hospital in Kielce, 25-736 Kielce, Poland; aga.chodala@vp.pl (A.C.-G.); grazyna.karolczyk@wszzkielce.pl (G.K.); 18 Department of Pediatrics, Oncology and Hematology, Medical University of Lodz, 91-738 Lodz, Poland; katarzynabobeff@gmail.com (K.B.); wojciech.mlynarski@umed.lodz.pl (W.M.); 19 Department of Pediatrics and Hematology and Oncology, Province Children’s Hospital, 10-561 Olsztyn, Poland; katarzynamycko@o2.pl (K.M.); hematologia@wssd.olsztyn.pl (W.B.); 20 Department of Pediatric Hematology and Oncology, Zabrze, Medical University of Silesia, 40-055 Katowice, Poland; rtomaszewska@szpital.zabrze.pl (R.T.); szczep57@poczta.onet.pl (T.S.); 21 Department of Pediatrics, Hematology and Oncology, City Hospital, 41-500 Chorzow, Poland; katmachnik@gmail.com; 22 Student Scientific Group of Pediatric Oncology and Hematology, Jagiellonian University Medical College, 30-663 Krakow, Poland; natalia.zamorska@student.uj.edu.pl

**Keywords:** FLT3, acute myeloid leukemia, children, kinase inhibitor

## Abstract

**Simple Summary:**

The FMS-like tyrosine kinase 3 (FLT3) gene mutated in 10–15% of pediatric acute myeloid leukemia (AML) is associated with an inferior outcome. We retrospectively analyzed the nationwide pediatric AML database from between 2005 and 2022. FLT3-ITD mutation was found in 10.7% of patients. An improvement in the outcome was found in the analyzed period of time. The treatment results in FLT3-ITD--positive patients treated under the AML-BFM 2012 and 2019 protocols were better in comparison to the AML-BFM 2004 protocol and better than previously reported by most authors. However, the outcome in patients with FLT3-ITD compared to children without this mutation was still significantly worse, with higher percentage of non-responders and relapses. It seems that SCT and FLT3 inhibitors have a beneficial impact on the prognosis; however, it should be confirmed in a larger group of patients. This gives hope for the improvement of the treatment results in pediatric AML with FLT3-ITD in the future.

**Abstract:**

Background: The FMS-like tyrosine kinase 3 (FLT3) gene mutated in 10–15% of pediatric acute myeloid leukemia (AML) is associated with an inferior outcome. The aim of the study was to analyze the outcome and characteristics of FLT3-ITD-positive pediatric AML. Methods: We retrospectively analyzed the nationwide pediatric AML database from between 2005 and 2022. FLT3-ITD was found in 54/497 (10.7%) patients with available analysis. Three consecutive treatment protocols were used (AML-BFM 2004 Interim, AML-BFM 2012 Registry, AML-BFM 2019 recommendations). Results: Probabilities of 5-year overall (OS), event-free (EFS) and relapse-free survival were significantly lower in the FLT3-ITD-positive patients compared to FLT3-ITD-negative (0.54 vs. 0.71, *p* = 0.041; 0.36 vs. 0.59, *p* = 0.0004; 0.47 vs. 0.70, *p* = 0.0029, accordingly). An improvement in the outcome was found in the analyzed period of time, with a trend of better survival in patients treated under the AML-BFM 2012 and AML-BFM 2019 protocols compared to the AML-BFM 2004 protocol (5-year EFS 0.52 vs. 0.27, *p* = 0.069). There was a trend of improved outcomes in patients treated with FLT3 inhibitors (n = 9, 2-year EFS 0.67 vs. 0.33, *p* = 0.053) and those who received stem cell transplantation (SCT) (n = 26; 5-year EFS 0.70 vs. 0.27, *p* = 0.059). The co-occurrence of the WT1 mutation had a dismal impact on the prognosis (5-year EFS 0.23 vs. 0.69, *p* = 0.002), while the NPM1 mutation improved survival (5-year OS 1.0 vs. 0.44, *p* = 0.036). Conclusions: It seems that SCT and FLT3 inhibitors have a beneficial impact on the prognosis. Additional genetic alterations, like the WT1 and NPM1 mutations, significantly influence the outcome.

## 1. Introduction

Acute myeloid leukemia (AML) is a biologically heterogeneous disease that comprises about 15–20% of pediatric acute leukemias. The prognosis of AML has improved significantly over the last few decades as a result of coordinated efforts through international research groups. However, despite the intensification of the treatment, more accurate stratification of the risk groups and the improvement in supportive care, around 20–30% of pediatric patients die from AML or its fatal complications [1,2].

FMS-like tyrosine kinase 3 (FLT3) is a transmembrane ligand-activated receptor tyrosine kinase. It is expressed on hematopoietic stem cells and early myeloid and lymphoid progenitor cells, and plays crucial role in the regulation of the proliferation, differentiation and apoptosis of hematopoietic cells [3,4,5]. FLT3 is mutated either by internal tandem duplications (FLT3-ITD) of the juxtamembrane domain or by a point mutation, usually involving the tyrosine kinase domain (TKD) [6]. FLT3 mutations occur in about 30% of adults with AML and about 10–15% of pediatric cases of AML [7,8,9]. The presence of FLT3-ITD is associated with poorer survival in both age groups due to the increased relapse rate, while FLT3-TKD mutations do not significantly impact the prognosis [6,7,10].

Taking into account the prognostic relevance of FLT3 mutations, they represent an appropriate therapeutic target [11,12,13]. FLT3 inhibitors have been studied for more than 20 years. They are categorized as type I (affecting both ITD and TKD mutations) or type II inhibitors (affecting only ITD mutation). Additionally, two generations of FLT3 inhibitors are distinguished. First-generation inhibitors have multikinase activity with a lower specificity for FLT3, while second-generation inhibitors are more specific for FLT3 activity [14]. The sorafenib a multikinase type II FLT3 inhibitor was one of the first to be studied in patients with AML. Its use has been analyzed in children with both newly diagnosed and relapsed/refractory AML with encouraging results [15,16]. Midostaurin, a first-generation type I FLT3 inhibitor, is the first to be FDA-approved for AML treatment in adults [17]. It is now being evaluated in clinical trials in children with newly diagnosed AML. Numerous newer generation FLT3 inhibitors with greater specificity to FLT3, and hopefully fewer off-target side effects, are still being evaluated in clinical studies [11]. The data on the effectiveness of the use of FLT3 inhibitors offer hope for their widespread use and for the improvement of the treatment results in AML with FLT3 mutations.

The aim of this study was the retrospective analysis of the clinical characteristics and treatment outcomes in children with AML and the FLT3-ITD mutation treated in Poland between 2005, when the analysis of FLT3 became widely available, and 2022.

## 2. Materials and Methods

**Figure 1:**
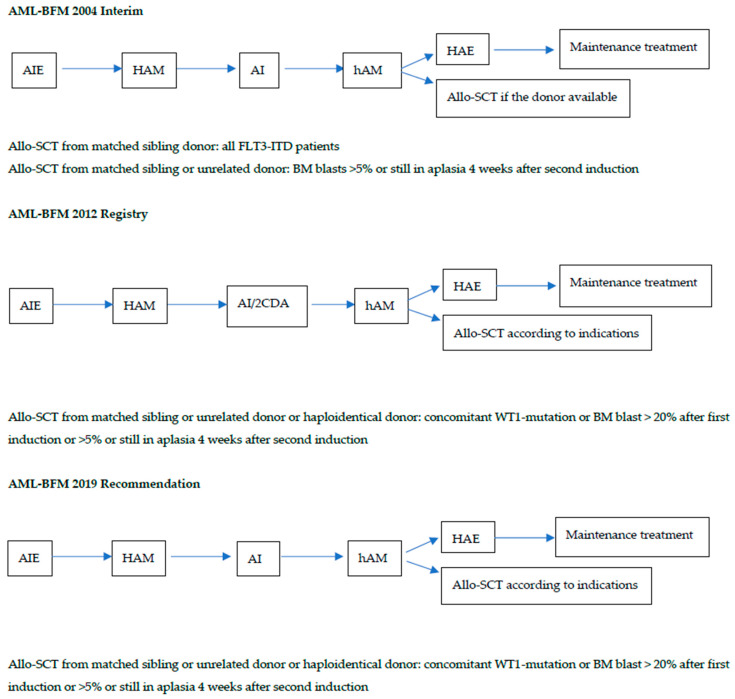
Treatment of FLT3-ITD-positive acute myeloid leukemia according to the consecutive protocols. A—cytarabine, I—idarubicine, E—etoposide, HA and hA—high dose cytarabine, M—mitoxantrone, Allo-SCT- allogenic stem cell transplantation, BM—bone marrow.

We performed retrospective analysis of the Polish Pediatric Leukemia and Lymphoma Study Group (PPLLSG) AML database. There were 577 patients with pediatric AML registered between 2005 and 2022, excluding acute promyelocytic leukemia (*n* = 54), myeloid leukemia in Down syndrome (*n* = 64), MDS-related AML (*n* = 37), AML post-cytotoxic therapy (*n* = 38) and biphenotypic leukemia (*n* = 20). Among the 497 children with available FLT3 analysis, FLT3-ITD was found in 54 patients (10.7%)—29 boys (53%) and 25 girls (47%), with a median age of 13.2 years (range: 3–17.9). Between 2005 and 2015, patients were treated under the AML-BFM 2004 Interim protocol (30 children); between 2015–2019, under the AML-BFM 2012 Registry (10 patients) and between 2019–2022, under the AML-BFM 2019 Recommendations (14 patients). According to AML-BFM 2004, all patients with FLT3-ITD mutation were stratified to the high risk group (HRG) and had indications for stem cell transplantation (SCT) from a matched sibling donor and in cases of non-response for SCT from a matched sibling or unrelated donor (Figure 1). In the protocols under the AML-BFM 2012 Registry and AML-BFM 2019, patients with FLT3-ITD were classified to the HRG with indications for SCT (from a sibling or unrelated or haploidentical donor) only if there was concomitant WT1 mutations or poor response to the treatment (Figure 1). Details of the treatment protocols are described elsewhere [18].

Nine patients (17%) received FLT3 inhibitors (8 patients sorafenib, 1 patient midostaurin) in addition to the standard first-line therapy, including 1 of 10 (10%) treated with AML-BFM 2012 and 8 of 14 patients (57%) treated with AML-BFM 2019. Four patients received FLT3 inhibitors as a part of the second-line therapy, including one who was also treated with a FLT3 inhibitor in the first-line therapy. FLT3 inhibitors were used on a compassionate-use basis; the AML-BFM 2019 Recommendations suggested use of Sorafenib in all FLT3-positive patients; the decision was made by the treating physician.

In 26 children (48%), SCT was performed in the first-line therapy, including 12/30 (40%) patients treated with AML-BFM 2004, 7/10 (70%) patients treated with AML-BFM 2012 and 7/14 (50%) patients treated with AML-BFM 2019.

In the group of patients treated with AML-BFM 2004 Interim, follow-up was completed on 31 December 2020, with a median observation time of 93 months (range: 29–157 months). The follow-up of the patients treated with AML-BFM 2012 Registry and AML-BFM 2019 Recommendations finished on 31 May 2023, with a median observation time of 29 months (range: 10–89 months).

Complete morphological remission was defined as less than 5% of blasts in BM demonstrating normal or only slightly decreased cellularity with signs of regeneration of normal hematopoiesis, regeneration of normal cell production in peripheral blood (≥1000/μL leukocytes, ≥500/μL neutrophil granulocytes and ≥50,000/μL platelets), lack of blasts in peripheral blood and the disappearance of any extramedullary sites. Minimal residual disease was assessed only in the patients treated with AML-BFM 2012 Registry and AML-BFM 2019 Recommendations as a part of a separate study; the results did not influence the therapeutic decision and were not analyzed in that paper.

Failure to achieve morphological remission under the first-line therapy was defined as no fulfillment of CR criteria by the end of the intensification or leukemic blasts ≥5% after 2nd induction or aplasia ≥6 weeks after the beginning of 2nd induction. Relapse was defined as reappearance of leukemic blasts in the peripheral blood, re-infiltration of BM with >5% blasts or leukemic infiltration elsewhere following CR or partial remission lasting at least 4 weeks.

Genetic analyses were performed centrally in the Department of Medical Genetics of University Children Hospital in Krakow. Between 2005 and 2018, the presence of FLT3-ITD was assessed through PCR amplification and the electrophoretic separation of products. The mutations in the WT1 and the NPM1 genes were detected through Sanger sequencing (WT1: DNA sequencing of exons 7 and 9 and NPM1: DNA sequencing of exons 10 and 11). Since 2019, pathogenic variants for all of the mentioned genes were detected through next-generation targeted sequencing (NGS) using The VariantPlex Core Myeloid panel (ARCHER - Integrated DNA Technologies, Inc. San Diego, California, USA). Statistical analyses were performed with STATISTICA, version 13, StatSoft Inc (Tulsa, OK, USA). Probabilities of survival were calculated using the Kaplan-Meier method. Overall-survival (OS) was defined as the time from the date of diagnosis to the date of death from any cause or the last follow-up. Event-free survival (EFS) was calculated as the time from diagnosis to the first event (relapse, death of any cause, failure to achieve remission or secondary malignancy) or the last follow-up. Failure to achieve morphological remission was considered an event on day 0. Relapse-free survival (RFS) was calculated as the time from the first remission to the relapse. RFS was analyzed only in patients who achieved CR. Probabilities of survival were presented as decimal fractions with standard deviations. The sub-groups were compared using the log-rank test. *p*-value less than 0.05 was considered statistically significant; values between 0.05 and 0.1 were considered a trend.

The study was conducted according to the guidelines of the Declaration of Helsinki, and approved by the Ethics Committee of Jagiellonian University (protocol code: 122.6120.17.2015, date of approval: 29 January 2015). Informed consent was obtained from all patients or guardians while entering into each therapeutic protocol.

## 3. Results

### 3.1. Comparison of the Patients with and without FLT3-ITD Mutation

The characteristics of the patients with FLT3-ITD and the comparison with the group without that mutation are presented in Table 1. The patients with FLT3-ITD, compared to the wild type (WT), were significantly older (13.4 (3.0–18.0) years vs. 8.8 (0.1–18.0), *p* < 0.0001), with higher initial leukocytosis (92 (6.5–573) × 109/L vs. 19 (0.9–979) × 109/L, *p* < 0.0001). The most common subtype, according to the FAB (French-American-British) classification, in the FLT3-ITD-positive group were M2 (37.7%) and M4 (22.2%), while there were no patients with the M6 or M7 subtype.

**Figure 2:**
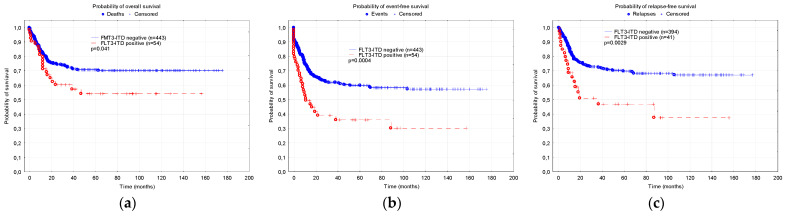
Comparison of survival in the group of patients with and without FLT3-ITD: (**a**) Overall survival; (**b**) Event free survival; (**c**) Relapse-free survival.

The probabilities of 5-year overall (OS), event-free (EFS) and relapse-free (RFS) survival were significantly lower in the FLT3-ITD-positive patients compared to the children without the mutation (0.54 ± 0.07 vs. 0.71 ± 0.03, *p* = 0.041; 0.36 ± 0.07 vs. 0.59 ± 0.03, *p* = 0.0004; 0.47 ± 0.09 vs. 0.70 ± 0.04, *p* = 0.0029, accordingly) (Figure 2).

The treatment results depending on FLT3-ITD are shown in Table 2.

In the group with the FLT3-ITD mutation, 41 patients (76%) achieved complete remission (CR) under the first-line therapy. There were 2 (3.7%) early deaths, 3 (5.5%) deaths in aplasia before CR, 8 (11%) non-responders, 19 patients (35%) relapsed, 1 (1.8%) patient died in CR and 16 (29.6%) died of disease progression, including 4 non-responders and 12 patients who relapsed. Twenty-five (46.3%) children remain in first CR (including four who achieved CR after second-line therapy), seven remain in second CR. Among the eight non-responders, three (37.5%) patients died of disease progression despite further treatment, five (62.5%) patients achieved CR on the second-line therapy, four (50%) of them are still in CR and one patient relapsed and died of the disease progression.

### 3.2. Characteristics of the Patients and Outcome Depending on the Treatment Protocol

The characteristics of the patients with FLT3-ITD depending on the treatment protocol are shown in Table 3. The median observation time was significantly longer in the patients treated with AML-BFM 2004 compared to the group treated with AML-BFM 2012 and AML-BFM 2019 (93.5 months vs. 28.7 months, *p* < 0.0001). A higher percentage of relapses (50% vs. 16.7%, *p* = 0.012) and a lower percentage of continuous remission (23.3% vs. 58.3%, *p* = 0.009) were observed in the patients treated with AML-BFM 2004 compared to the group treated under the consecutive protocols.

**Figure 3:**
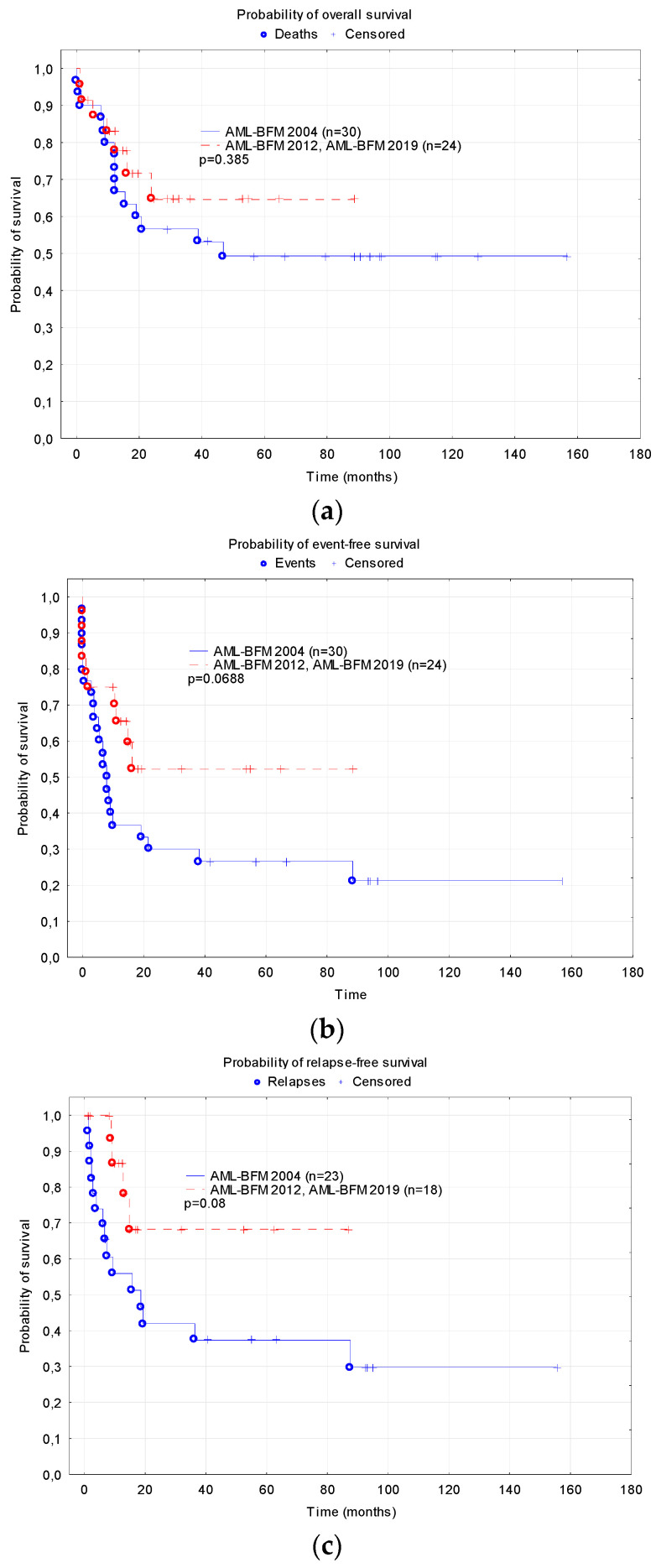
Probability of survival depending on the treatment protocols (AML-BFM 2004 Interim vs. AML-BFM 2012 Registry and AML-BFM 2019 recommendation): (**a**) Overall survival; (**b**) Event free survival; (**c**) Relapse-free survival.

There was a trend of a higher probability of EFS and RFS in the patients treated under the AML-BFM 2012 and 2019 protocols compared to the group treated under the AML-BFM 2004 protocol (5-year EFS 0.52 ± 0.12 vs. 0.27 ± 0.08, *p* = 0.0688; RFS 0.68 ± 0.13 vs. 0.37 ± 0.10, *p* = 0.08, Figure 3). The probability of 5-year OS did not differ significantly depending on the treatment protocols (0.65 ± 0.11 vs. 0.49 ± 0.09, *p* = 0.385).

### 3.3. FLT3-Inhibitors

**Figure 4:**
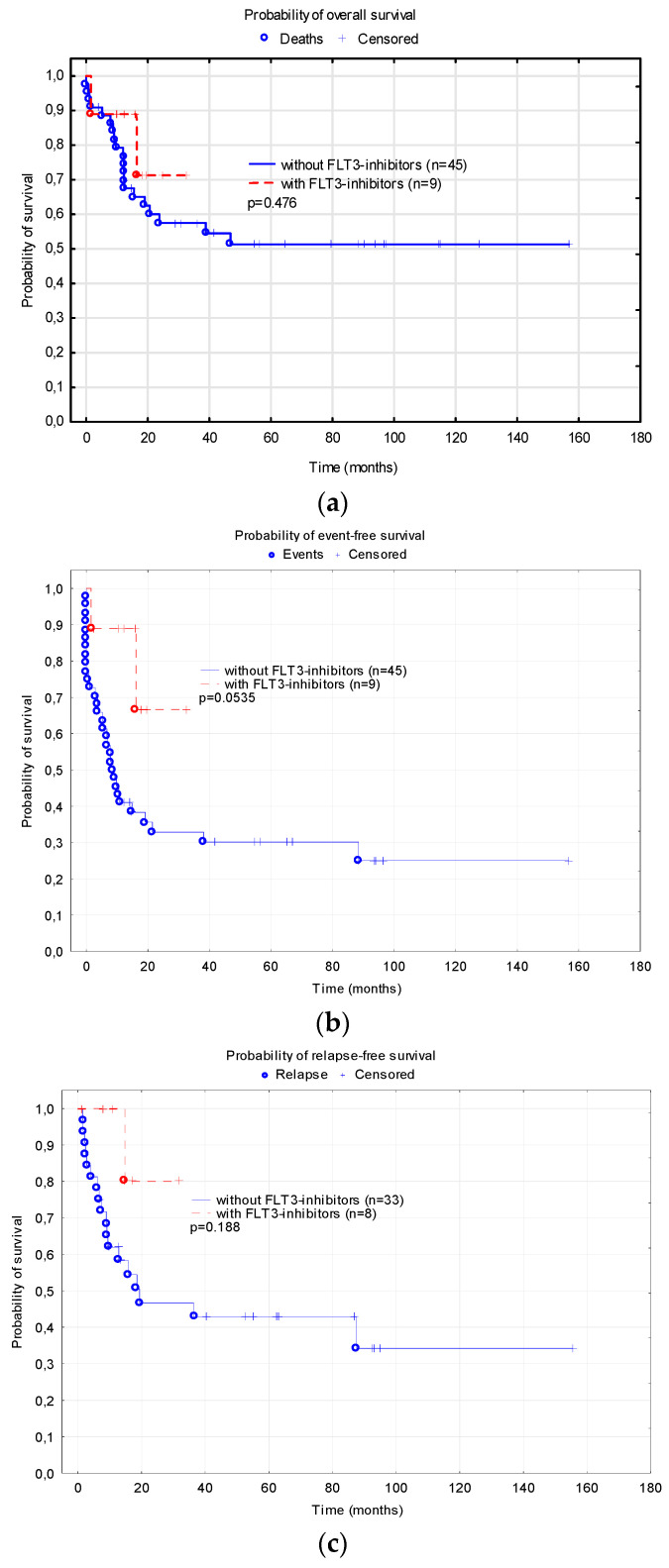
Probability of survival depending on the use of FLT3 inhibitors: (**a**) Overall survival; (**b**) Event free survival; (**c**) Relapse-free survival.

In the group of nine patients treated with FLT3 inhibitors (eight with sorafenib, one with midostaurin) in the first-line therapy, the probability of 2-year EFS was almost significantly higher than in the patients with FLT3-ITD who did not receive FLT3 inhibitors (0.67 ± 0.20 vs. 0.33 ± 0.07, *p* = 0.0535). There were no significant differences in the 2-year OS (0.71 ± 0.18 vs. 0.57 ± 0.08, *p* = 0.476) and RFS (0.80 ± 0.18 vs. 0.47 ± 0.09, *p* = 0.188) (Figure 4).

### 3.4. Stem Cell Transplantation

**Figure 5:**
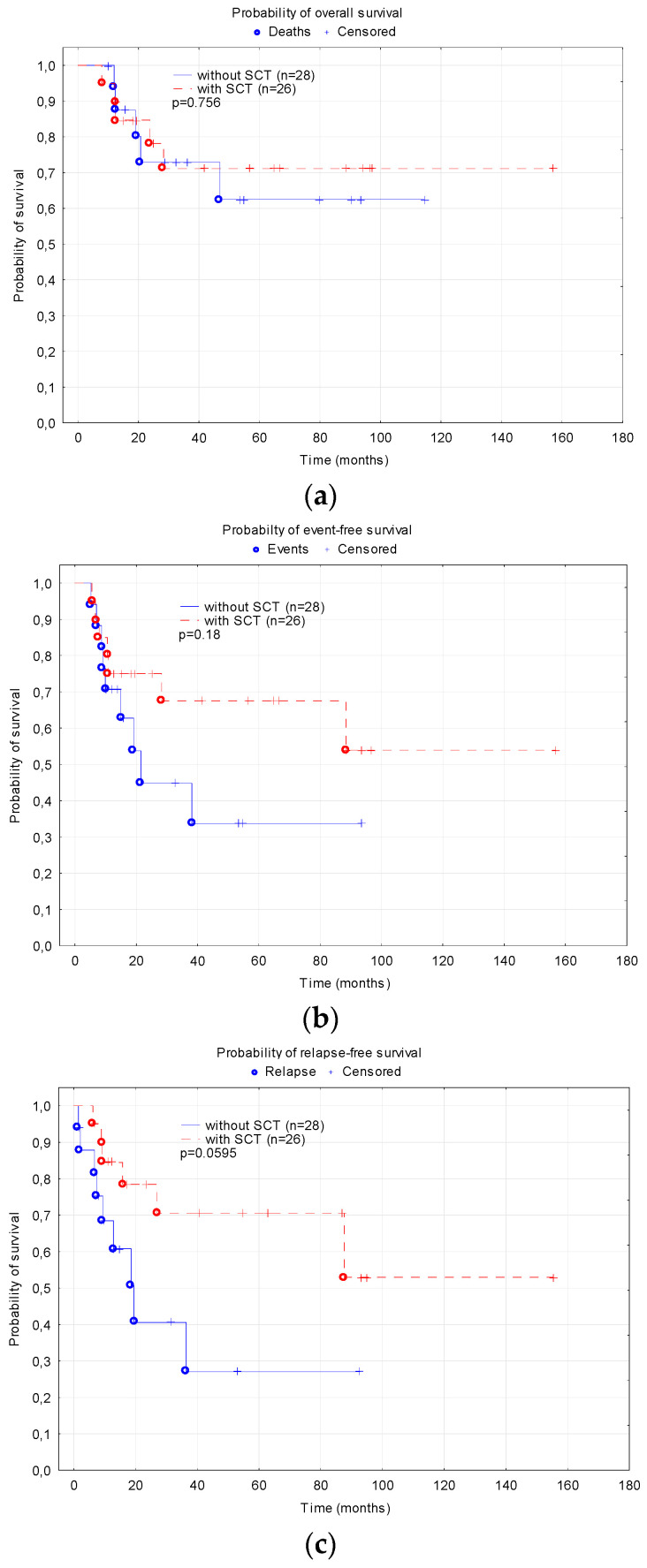
Comparison of survival in the group of patients treated with stem cell transplantation (SCT) and without SCT: (**a**) Overall survival; (**b**) Event free survival; (**c**) Relapse-free survival.

The analysis of the impact of SCT on survival included patients with an event-free survival of at least 4 months (HSCT was performed after at least four chemotherapy cycles). There was a trend toward a higher probability of RFS in transplanted patients compared to those who did not receive SCT (0.70 ± 0.11 vs. 0.27 ± 0.15, *p* = 0.0595, Figure 5). The probabilities of OS and EFS did not differ significantly depending on SCT (0.7 ± 0.11 vs. 0.62 ± 0.14, *p* = 0.756 and 0.67 ± 0.11 vs. 0.34 ± 0.14, *p* = 0.18).

### 3.5. Treatment Results Depending on Additional Mutation

**Figure 6:**
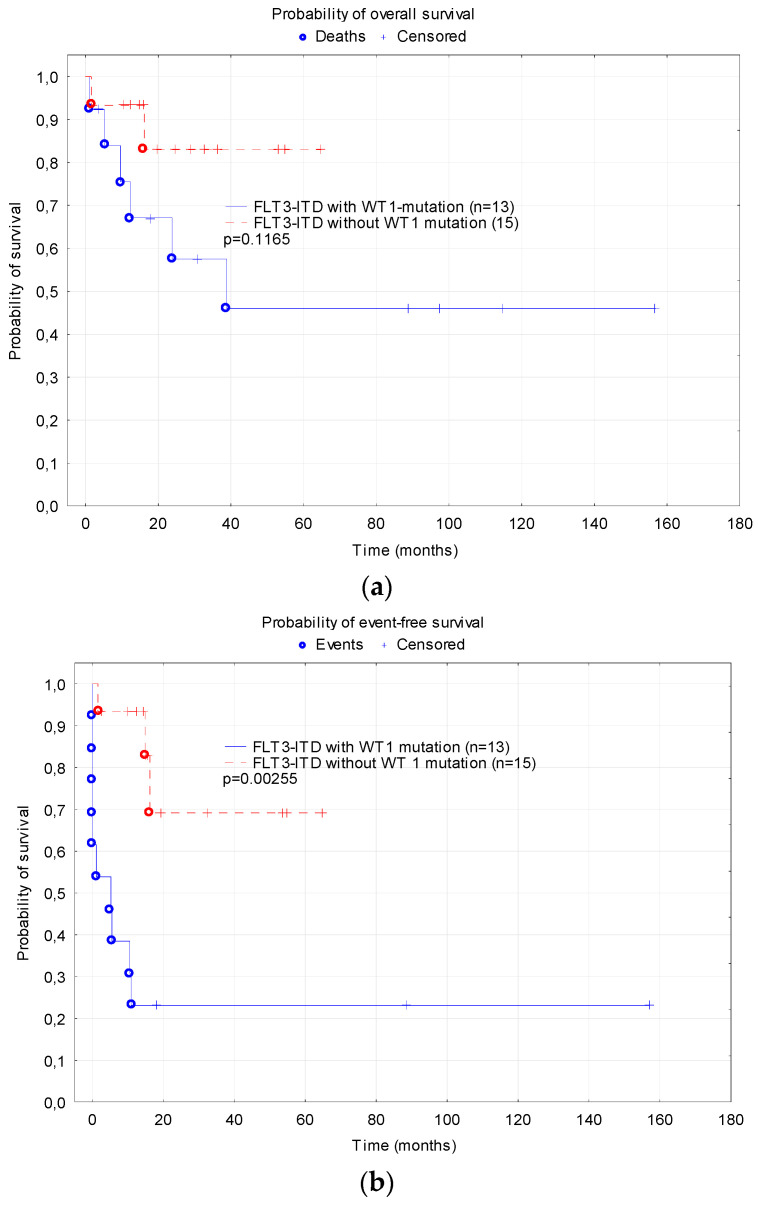

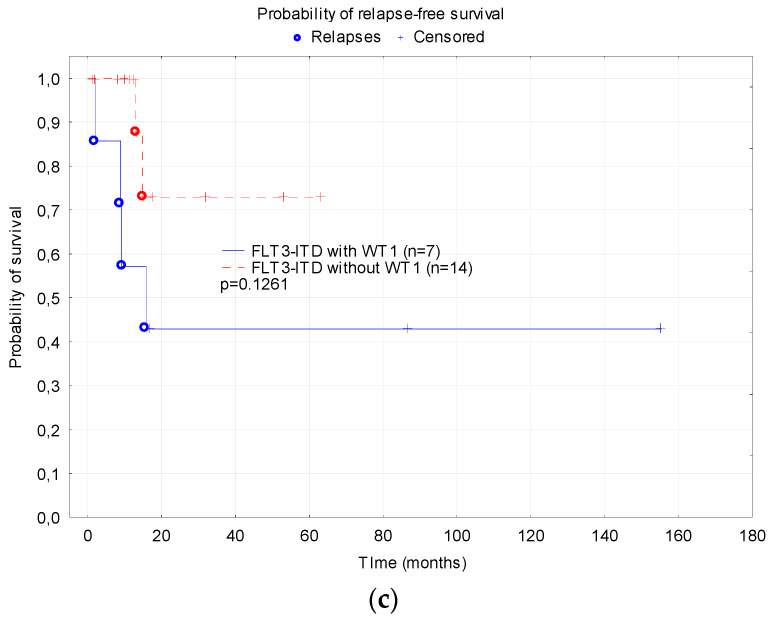
Probability of survival depending on WT1 mutation: (**a**) Overall survival; (**b**) Event free survival; (**c**) Relapse-free survival.

The analysis of the WT1 mutation was performed in the patients treated with AML-BFM 2012 and 2019. The WT1 mutation was co-expressed in 13 of the 28 FLT3-ITD-positive patients (46%) with available results, including one patient with both the WT1 and NPM1 mutations. In that group, seven (53.8%) patients achieved CR, there were five (38.5%) non-responders and one (7.7%) early death occurred. Four patients (30.7%) relapsed. There were five (38.5%) deaths caused by disease progression, including three non-responders and two patients after relapse. Five patients (38.5%) remain in the first CR, including two who achieved CR after second-line therapy. Two patients remain in the second CR. The patients with a co-expression of the WT1 mutation had a significantly lower probability of 2-year EFS compared to the children with FLT3-ITD without the WT1 mutation (0.23 ± 0.12 vs. 0.69 ± 0.16, *p* = 0.0025) (Figure 6). In the group of patients with FLT3-ITD and the WT1 mutation, 11/13 of the children (85%) were transplanted in the first CR (one patient relapsed 2 months after remission before transplantation and was transplanted in the second remission, one patient died of toxicities before remission achievement). Among the 15 patients with FLT3-ITD without the WT1 mutation, six (40%) were transplanted, and there was no difference in the survival in that group depending on SCT.

**Figure 7:**
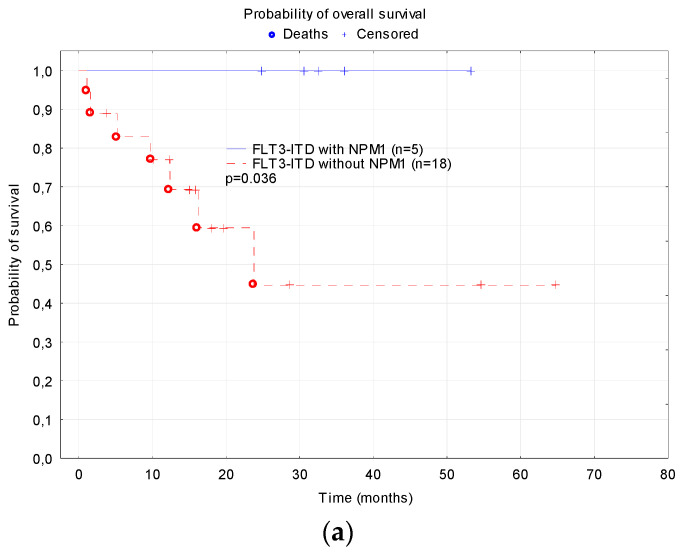

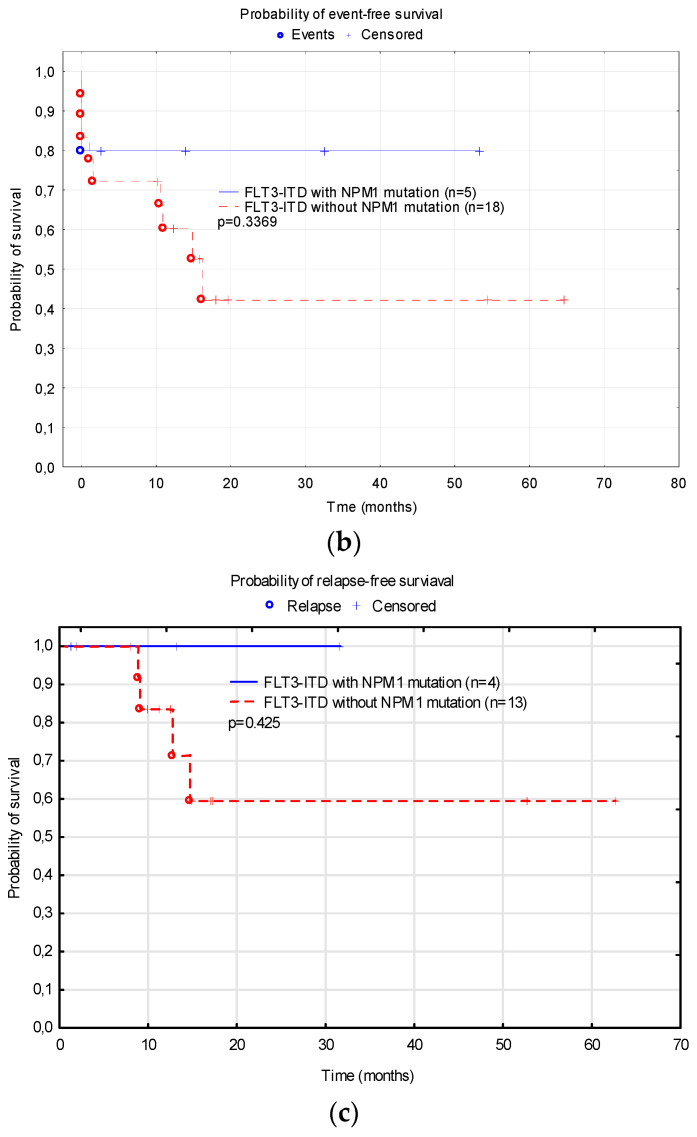
Probability of survival depending on NPM1 mutation: (**a**) Overall survival; (**b**) Event free survival; (**c**) Relapse-free survival.

The analysis of the NPM1 mutation was performed in the patients treated with AML-BFM 2012 and 2019. The co-expression of the NPM1 mutation with FLT3-ITD was found in 5 of the 23 children (22%) with available results, including one patient with both the WT1 and NPM1 mutation. In that group, four (80%) patients achieved CR on the first-line therapy, and one of them was transplanted. There was one non-responder (the patient with both NPM1 and WT1 mutations), who achieved remission on the second-line treatment and was then transplanted. No death occurred in the group of patients who harbored both FLT3-ITD and the NPM1 mutation. All of the patients remain in CR. The co-expression of the NPM1 mutation was associated with significantly higher 2-year OS compared to the patients with FT3-ITD without NPM1 (1.0 vs. 0.44 ± 0.16, *p* = 0.036) (Figure 7).

## 4. Discussion

In the present study, FLT3-ITD was found in 10.7% of the children with AML. This is similar the rates reported in other studies concerning pediatric AML (10.3–21%) [8,9,19,20,21,22], while in adults, the frequency of the FLT3 mutation is rather higher (25–30%) [22,23]

We found that the patients with FLT3-ITD were older and had a higher number of leukocytes at diagnosis compared to the FLT3-ITD-negative group. This was in accordance with other studies [8,19,22].

The most common subtypes, according to the FAB classification, in the FLT3-ITD-positive group were M2 (37.7%) and M4 (22.2%); there were no patients with the M6 or M7 subtype. This is similar to the results described before [8,19,22].

In the present study, the analysis of concomitant genetical abnormalities in the group with FLT3-ITD revealed that most of the patients did not have any fusion genes; KMT2A rearrangements were rare, while DEK::NUP214 was significantly more frequent than in patients with FLT3-WT. Shimada et al. reported that FLT3-ITD-positive patients presented more often with normal, t(6;9)(p23;q34) and +8 karyotypes and less frequently exhibited t(8;21)(q22;q22) or inv(16)(p13q22) karyotypes compared to FLT3-ITD WT patients [22]. Similarly, Semary et al. observed that in the group with FLT3-ITD compared to FLT3-ITD WT, patients with normal karyotype and t(6;9)(p23;q34) were more frequent, while favorable cytogenetics (t(8;21) and inv(16)) were less common [19].

The treatment results regarding AML with FLT3-ITD in the current study are comparable to the results reported by other authors. In our cohort, the 5-year OS, EFS and RFS were 0.54 ± 0.07l; 0.36 ± 0.07 and 0.47 ± 0.09, respectively. In a study from 2003, Zwaan et al. showed a CR rate of 70% and 5-year OS, EFS and DFS of 32%, 29% and 41%, respectively, in a group of 27 children with FLT-ITD treated between 1990 and 2001 within an AML-BFM study group and DCLSG. Ostronoff et al. analyzed a group of 253 adults and children with FLT3-ITD enrolled in five consecutive Children’s Oncology Group/Children’s Cancer Group and SWOG trials, and observed a 3-year OS of 45% and EFS of 30% [20]. Shimada et al. reported the results from the JPLSG AML-05 study and showed that in the group of 47 patients with FLT-ITD, the 5-year OS, EFS and DFS were 42.2%, 36.8% and 58.4%, respectively [22].

In the current study, the patients were treated according to three consecutive protocols (AML-BFM 2004 Interim, AML-BFM 2012 Registry and AML-BFM 2019 Recommendations). According to the AML-BFM 2004 Interim (first period) protocol, all patients with FLT3-ITD were classified into the high-risk group and were intended to receive SCT from a matched sibling donor, or in cases of non-response, also from a matched unrelated donor. Taking into account the proven influence of concomitant mutations on the outcome, the presence of the WT1 and NPM1 mutations was included in the stratification of the risk groups in the consecutive protocols (AML-BFM 2012 and AML-BFM 2019—second period). According to those protocols, patients with FLT3-ITD were classified in the HRG, and consequently to SCT (sibling or unrelated or haploidentical donor), only if there was a co-expression of the WT1 mutation or poor response to the induction therapy. The percentage of transplanted patients was higher in the group treated according to the AML-BFM 2012 and AML-BFM 2019 (58.3%) protocols compared to children treated with AML-BFM 2004 (40%). This was probably due to the lack of acceptance of matched unrelated donors for HRG patients responding to the first-line treatment according to the AML-BFM 2004 Interim protocol. A higher percentage of relapses and a lower percentage of continuous remission were observed in patients treated with AML-BFM 2004 Interim compared to the group treated with the consecutive protocols. The lower incidence of relapses in the second period could be partially explained by the shorter follow-up compared to the first period (29 months vs. 93 months); however, most relapses occurred within the first 2 years from AML diagnosis. As there were still some patients censored before 2 years from diagnosis, further observations will be crucial in order to draw final conclusions. There was a trend of a higher probability of EFS and RFS in patients treated with the AML-BFM 2012 and 2019 protocols compared to the group treated with the AML-BFM 2004 protocol (5-year EFS 0.52 vs. 0.27, *p* = 0.0688; RFS 0.68 vs. 0.37, *p* = 0.08), while the probability of 5-year OS did not differ significantly depending on the treatment protocols (0.65 vs. 0.49, *p* = 0.385). The results of the treatment of pediatric AML with FLT3-ITD in the second period (protocols AML-BFM 2004 Interim and AML-BFM 2012 Registry) in our study were better than those previously described [8,19,20,22]. The superior survival in the second period may be due to the higher percentage of transplanted patients, especially those at the highest risk of treatment failure (concomitant WT1 mutation), and the use of FLT3 inhibitors. Moreover, the improvement to supportive care may have influenced the survival; however, the percentages of early deaths and deaths in remission did not differ significantly. In the current study, there was a trend toward a higher probability of RFS in transplanted patients compared to those who did not receive SCT (0.70 vs. 0.27, *p* = 0.0595), while the probabilities of OS and EFS did not differ significantly depending on SCT. The lack of statistical significance could be due to the small size of the group (26 patients who underwent SCT). Semary et al. reported significantly higher probabilities of OS and EFS in patients with FLT3-ITD who received SCT (77.8% and 78.8%, respectively) compared to those who were treated with chemotherapy alone (16.3% and 12.8%, *p* < 0.001); however, it seems that the authors did not exclude early deaths, induction failures and early relapses from the analysis [19]. In the study by Zwaan et al., multivariate analysis did not show any prognostic benefit of SCT in the first CR in patients with FLT3-ITD [8], while Niktoreh et al. found that SCT significantly improved the EFS (HR 0.25, *p* = 0.0004) but did not influence the OS [24].

In our study, in the first period (protocol AML-BFM 2004 Interim), no patients received FLT3 inhibitors, while there were nine patients (37.5%) in the second period who were treated with sorafenib or midostaurin in addition to the standard chemotherapy. We have found that the use of FLT3 inhibitors improved the 2-year EFS (0.67 vs. 0.33, *p* = 0.0535), while there was no significant impact on the OS and RFS. The results of the analysis may not be significant due to the small number of patients treated with FLT3 inhibitors (n = 9). There are numerous studies on FLT3 inhibitors; however, most of them concern the adult population. The preliminary results of clinical trials demonstrate that the addition of sorafenib increases the CR and EFS and decreases the relapse risk in children with high AR FLT3-ITD [14,16]. A retrospective study showed that sorafenib induced remissions in children with relapsed FLT3/ITD+ AML and long-term maintenance significantly improved the OS [25]. According to the recent expert recommendation, post-transplant FLT3 inhibitors should be administered for children with FLT3-ITD AML up to 12–24 months post-HSCT [26]. Midostaurin is the first FDA-approved FLT3 inhibitor. The multinational RATIFY study on adult patients demonstrated that the addition of midostaurin to standard chemotherapy with SCT prolonged the survival and decreased the relapse risk compared to chemotherapy and SCT alone [17]. A phase II study of midostaurin with chemotherapy in untreated, newly diagnosed, FLT3-mutated pediatric AML is ongoing. Another second-generation type I FLT3 inhibitor, gilteritinib, is FDA-approved in adults with relapsed/refractory FLT3-mutated AML [14]. The retrospective data of eight pediatric patients published by McCall et al. revealed a 62% remission rate [27]. Two ongoing pediatric trials will investigate the use of gilteritinib in FLT3-mutated AML in combination with chemotherapy in relapsed disease and as upfront therapy [14,27].

The prognostic impact of concomitant genetic alterations such as WT1, NPM1 and NUP98/NSD1 has been studied by many authors before. The co-occurrence of the WT1 mutation and/or NUP98/NSD1 with FLT3-ITD has been proven to be a poor prognostic factor [20,22,24,28], while the results of the studies concerning the role of the NPM1 mutation are ambiguous [22,24,28,29].

Unfortunately, NUP98/NSD1 was not assessed in our cohort.

Analysis of the WT1 mutation was available in 28 patients in the current study. The co-occurrence of the WT1 mutation was found in 46% of the patients and was associated with a poor outcome (5-year EFS 0.23 vs. 0.69, *p* = 0.0025), with a high percentage of non-responders (38.5%). Similarly, it was described in the study by Shimada et al., who found that the WT1 mutation affected the EFS (HR 4.16, *p* = 0.04) [22]. Niktoreh et al. analyzed the role of mutations in WT1 and FLT3-ITD as prognostic factors in two contemporary pediatric treatment protocols in the AML-BFM study group. They found that the presence of both mutated WT1 and FLT3-ITD (n = 19) resulted in poorer survival (3-year EFS and OS of 29% and 33% compared 45–63% and 67–87% in patients with only FLT3-ITD (n = 33), only WT1 mutation (n = 29) or none of these mutations (n = 272)) [24]. Importantly, we have shown that in patients with FLT3-ITD without the WT1 mutation, SCT does not influence the outcome. This confirms the validity of the risk group stratification in the AML-BFM 2012 and 2019 protocols, where patients with FLT3-ITD without WT1 mutations are not referred to SCT in cases of a good response to the treatment.

In the present study, the NPM1 mutation was revealed in 5 of the 23 (22%) FLT3-ITD patients with available NPM1 analysis. This small group of children was characterized by an excellent outcome, with no deaths and no relapses, even though most of them were not transplanted. Despite the very small size of this subgroup, the results of the treatment in the patients with concomitant FLT3-ITD and NPM1 mutation in our study are in accordance with the data published by Shimada et al., who revealed that all five patients with FLT3-ITD and co-occurrent NPM1 mutation survived [22]. In addition, Hollink who showed that NPM1 mutations were an independent favorable prognostic factor on the EFS (80% vs. 39%; *p* = 0.01) and OS (85% vs. 60%; *p* = 0.06), which was not influenced by FLT3-ITD [29]. Interestingly, Niktoreh et al. did not find an impact of the NPM1 mutation on survival in pediatric patients with FLT3-ITD [24].

The prognostic relevance of the FLT3-ITD allelic ratio (AR) has been studied widely. While previous trials demonstrated that an AR > 0.4 was associated with poorer prognosis compared to an AR < 0.4 [7,8], updated analysis has suggested that an AR > 0.1 may also significantly impact survival [14,30]. Unfortunately, the FLT3-ITD allelic ratio was not assessed in our cohort.

The main limitation of the study is its retrospective nature. Moreover, the small number of patients in the subgroups renders statistical analysis difficult. In addition, no allelic-ratio assays or MRD analysis were available in the current study. Nevertheless, given the low incidence of AML with the FLT3 mutation, our retrospective study of 54 children treated over the last 18 years across Poland seems to be valuable, especially as it also includes treatment with FLT3 inhibitors.

## 5. Conclusions

To conclude, in the analyzed group of pediatric AML patients, the FLT3-ITD mutation was found in 10.7% of the patients. The treatment results in children with AML and the FLT3-ITD mutation in the current study were similar to those described by other authors. An improvement in the outcome was found in the analyzed period of time. The treatment results in the second period (AML-BFM 2012 and 2019 protocols) were better in comparison to the AML-BFM 2004 protocol, and better than those previously reported by most authors. However, the outcomes in patients with FLT3-ITD compared to the children without this mutation were still significantly worse, with a higher percentage of non-responders and relapses. It seems that SCT and FLT3 inhibitors have a beneficial impact on the prognosis; however, this should be confirmed in a larger group of patients. This gives hope for the improvement of the treatment results in pediatric AML with FLT3-ITD in the future.

## Figures and Tables

**Table 1:** Characteristics of the patients depending on the presence of FLT3-ITD.

	FLT3 WT	FLT3-ITD	*p*
Number of patients	443	54	
Age median (range) years	8.8 (0.1–18.0)	13.4 (3.0–18.0)	<0.0001
Gender male/female	229/214	29/25	NS
WBC at diagnosis—median (range) ×10^9^/L	19 (0.9–979)	92 (6.5–573)	<0.0001
FAB types			
M0	23 (5.2%)	4 (7.4%)	NS
M1	58 (13.1%)	9 (16.7%)	NS
M2	109 (24.6%)	20 (37.0%)	NS
M4	71 (16.0%)	12 (22.2%)	NS
M5	118 (26.6%)	8 (14.8%)	0.044
M6	6 (1.3%)	0	NS
M7	35 (7.9%)	0	0.029
Non defined	23 (5.2%)	1 (1.8%)	
Fusion genes analysis	N = 419	N = 48	
No fusion genes	216 (51.5%)	36 (75%)	0.002
RUNX1::RUNX1T1	76 (18.1%)	0	0.0013
CBFB::MYH11	28 (6.7%)	3 (6.2%)	NS
MLL::MLLT3	27 (6.4%)	0	NS
MLL::MLLT10	27 (6.4%)	0	NS
MLL::MLLT1	9 (2.1%)	0	NS
MLL::MLLT4	4 (0.9%)	1 (2%)	NS
MLL::MLLT6	3 (0.7%)	0	NS
DEK::NUP214	6 (1.4%)	4 (8.3%)	0.0018
BCR::ABL	3 (0.7%)	0	NS
Others	20 (4.8%)	2 (4.2%)	NS
Additional mutations			
WT1	15/189 (7.9)	13/28 (46.4)	<0.0001
NPM1	4/180 (2.2)	5/23 (21.7)	0.0002
Complex karyotype	40/370 (10.8%)	2/40 (5%)	NS

FLT3—FMS-like tyrosine kinase, ITD—internal tandem duplication, WT—wild type, NS—no significant difference, WBC—number of white blood cells.

**Table 2:** Treatment results depending on the presence of FLT3-ITD.

	FLT3 WT	FLT3-ITD	*p*
Number of patients	443	54	
CR (%)	394 (88.9)	41 (76)	0.0063
Early deaths (%)	10 (2.2)	2 (3.7)	NS
Deaths in aplasia before CR (%)	7 (1.6)	3 (5.5)	0.049
Non-responders (%)	24 (5.4)	8 (11)	0.008
Relapses (%)	101 (22.7)	19 (35)	0.045
Deaths in CR (%)	18 (4.1)	1 (1.8)	NS
Deaths from disease progression (%)	58 (13.1)	16 (29.6)	0.013
Continuous CR after first-line therapy (%)	275 (62.1)	21 (38.9)	0.001
5-year OS	0.71 ± 0.03	0.54 ± 0.07	0.041
5-year EFS	0.59 ± 0.03	0.36 ± 0.07	0.0004
5-year RFS	0.70 ± 0.04	0.47 ± 0.09	0.0029

FLT3—FMS-like tyrosine kinase, ITD—internal tandem duplication, WT—wild type, CR—complete remission, OS—overall survival, EFS—even-free survival, RFS—relapse-free survival.

**Table 3:** Characteristics of the patients with FLT3-ITD depending on the treatment protocol.

Protocol	AML-BFM 2004 Interim	AML-BFM 2012 Registry AML-BFM 2019 Recommendations	*p*
Period	2004–2014	2015–2022	-
Follow-up end-point	31 December 2020	31 May 2023	-
Median observation time (range) months	93.5 (29–156.8)	28.7 (10.2–88.6)	<0.0001
Number of patients	30	24	-
Age median (range) years	12.8 (3.0–17.7)	13.4 (6.9–17.9)	NS
Gender male/female	18/12	11/13	NS
FAB types (%)			-
M0	3 (10)	1 (4.2)	NS
M1	8 (26.7)	1 (4.2)	0.029
M2	9 (30)	12 (50)	NS
M4	7 (23.3)	5 (20.8)	NS
M5	3 (10)	5 (20.8)	NS
M6	0	0	-
M7	0	0	-
Fusion genes	N = 24	N = 23	
CBFB::MYH11	2 (8.3)	1 (4.3)	NS
MLL::MLLT4	0	1 (4.3)	NS
DEK:NUP214	0	4 (17.4)	NS
Risk groups (%)			-
SR	0	0	-
IR	-	8 (33.3)	-
HR	30 (100)	16 (66.7)	NS
Treatment results			
CR	23 (76.7)	18 (75)	NS
Early deaths (%)	2 (6.7)	0	NS
Deaths in aplasia before CR (%)	1 (3.3)	2 (8.3)	NS
Non-responders (%)	4 (13.3)	4 (16.7)	NS
Relapses (%)	15 (50)	4 (16.7)	0.012
Deaths in CR (%)	1 (3.3)	0	NS
Deaths from disease (%)	11 (36.7)	5 (20.8)	NS
Continuous CR (%)	7 (23.3)	14 (58.3)	0.009
SCT (%)	12 (40)	14 (58.3)	NS
5-year OS	0.49 ± 0.09	0.65 ± 0.11	NS
5-year EFS	0.27 ± 0.08	0.52 ± 0.12	0.069
5-year RFS	0.37 ± 0.10	0.68 ± 0.13	0.08

NS—no significant difference, SR—standard risk, IR—intermediate risk, HR—high risk, CR—complete remission, SCT—stem cell transplantation, OS—probability of overall survival, EFS—probability of event-free survival, RFS—probability of relapse-free survival.

## Data Availability

The data presented in this study are available on request from the corresponding author. The data are not publicly available due to privacy and ethical restrictions.
